# Case Report: Durable response to tumor-infiltrating lymphocyte therapy in a patient with metastatic melanoma and chronic lymphocytic leukemia/small lymphocytic lymphoma

**DOI:** 10.3389/fimmu.2025.1718443

**Published:** 2025-11-17

**Authors:** Lilit Karapetyan, Joel Kuriakose, Matthew C. Perez, Johannes R. Ali, MacLean S. Hall, Matthew S. Beatty, Christopher D. Otteni, Jin Xu, Denise Kalos, Carlos Moran-Segura, Ella Vieira, Karla Adams, Lianicet Rodriguez-Alfonso, Elizabeth C. DiMaggio, Hanna Bailey, Javier Pinilla-Ibarz, Frederick L. Locke, John E. Mullinax, Pei-Ling Chen, Jinming Song, Jane L. Messina, Douglas B. Johnson, David S. Morgan, Zeynep Eroglu, Nikhil I. Khushalani, Vernon K. Sondak, Amod A. Sarnaik, Kenneth Tsai, James J. Mulé, Shari Pilon-Thomas

**Affiliations:** 1 Department of Cutaneous Oncology, H. Lee Moffitt Cancer Center and Research Institute, Tampa, FL, United States; 2 Department of Biomedical Sciences, University of South Florida, Tampa, FL, United States; 3 Department of Immunology, H. Lee Moffitt Cancer Center and Research Institute, Tampa, FL, United States; 4 Department of Diagnostic Imaging, H. Lee Moffitt Cancer Center and Research Institute, Tampa, FL, United States; 5 Department of Biostatistics and Bioinformatics, H. Lee Moffitt Cancer Center and Research Institute, Tampa, FL, United States; 6 Department of Pathology, H. Lee Moffitt Cancer Center and Research Institute, Tampa, FL, United States; 7 Department of Blood and Marrow Transplant and Cellular Immunotherapy, H. Lee Moffitt Cancer Center and Research Institute, Tampa, FL, United States; 8 Sarcoma Department, H. Lee Moffitt Cancer Center and Research Institute, Tampa, FL, United States; 9 Department of Medicine, Vanderbilt University Medical Center and Vanderbilt Ingram Cancer Center, Nashville, TN, United States

**Keywords:** melanoma, tumor infiltrating lymphocytes, chronic lymphocytic leukemia/small lymphocytic lymphoma, cell therapy, adoptive cell immunotherapy

## Abstract

Tumor-infiltrating lymphocyte (TIL) therapy has demonstrated efficacy for the treatment of immune checkpoint therapy-resistant/refractory advanced melanoma patients. However, its safety and efficacy remain to be determined among patients with concurrent chronic lymphocytic leukemia (CLL)/small lymphocytic lymphoma (SLL) and advanced melanoma. This case is the first to demonstrate the successful treatment of a metastatic melanoma patient with concurrent CLL/SLL using lymphodepleting chemotherapy followed by unselected TIL and interleukin-2. The treatment was safe with no new toxicities observed, resulting in a durable radiological partial response and molecular response with the complete clearance of detectable circulating tumor DNA. The microenvironment of the harvested melanoma used to generate the TIL product contained an abundance of CD8^+^TCF1^+^ and CD8^+^CD69^+^ T cells, spatial co-clustering of CD8^+^ T cells with CD11c myeloid networks. Despite containing SLL deposits, the harvested melanoma produced a CD8^+^ T cell-predominant tumor-reactive TIL infusion product containing stem-like CD39^neg^CD69^neg^ CD8^+^ T cells. A strong overlap (60%) in the TCRβ sequences between this infusion product and peripheral blood mononuclear cells collected at the 9-month follow-up visit was evidence of long-term persistence of TIL in this patient.

## Background

Adoptive cell therapy (ACT) with tumor-infiltrating lymphocytes (TILs) has emerged as a promising approach for the management of metastatic melanoma (1–3). TIL-ACT utilizes a lymphodepleting non-myeloablative chemotherapy regimen that consists of cyclophosphamide and fludarabine, followed by *ex vivo* expanded autologous TIL infusion and high-dose interleukin-2 (IL-2). The combination of these three components has resulted in the improved persistence and functionality of adoptively transferred TIL. Lifileucel (Amtagvi^®^, Iovance Biotherapeutics, San Carlos, CA, USA) is an autologous unselected TIL product, which was most recently Food and Drug Administration (FDA)-approved as a standard-of-care therapy in patients with advanced melanoma who were previously treated with a programmed death-1 (PD-1) blocking antibody and a BRAF inhibitor +/− MEK inhibitor if *BRAF V600* mutation-positive. In a phase 2 study in 153 heavily pretreated patients with advanced melanoma, lifileucel demonstrated an objective response rate (ORR) of 31.4%, median progression-free survival (PFS) of 4.1 months, and median overall survival of 13.9 months with a safety profile generally associated with known toxicities due to lymphodepleting chemotherapy and IL-2 (1). The effectiveness of TIL therapy was further evaluated in a randomized clinical trial of patients with anti-PD-1-resistant/refractory advanced melanoma and demonstrated improved PFS in comparison to ipilimumab only (HR = 0.50) (4). Notwithstanding, the safety and efficacy of TIL have, to date, not been evaluated in patients with concurrent metastatic melanoma and chronic lymphocytic leukemia (CLL)/small lymphocytic lymphoma (SLL), as those patients have been historically excluded from initial clinical trials. Response to TIL among those patients may be compromised due to potential contamination of melanoma procurement tissue with CLL/SLL deposits, leading to immune dysregulation in the tumor microenvironment. Here, we report a patient with advanced melanoma and CLL/SLL who successfully completed TIL therapy and the evaluation of the immune microenvironment of the harvested melanoma for TIL production, as well as the immunophenotypic characteristics of the TIL product related to the clinical melanoma response.

## Case presentation

**Figure 1:**
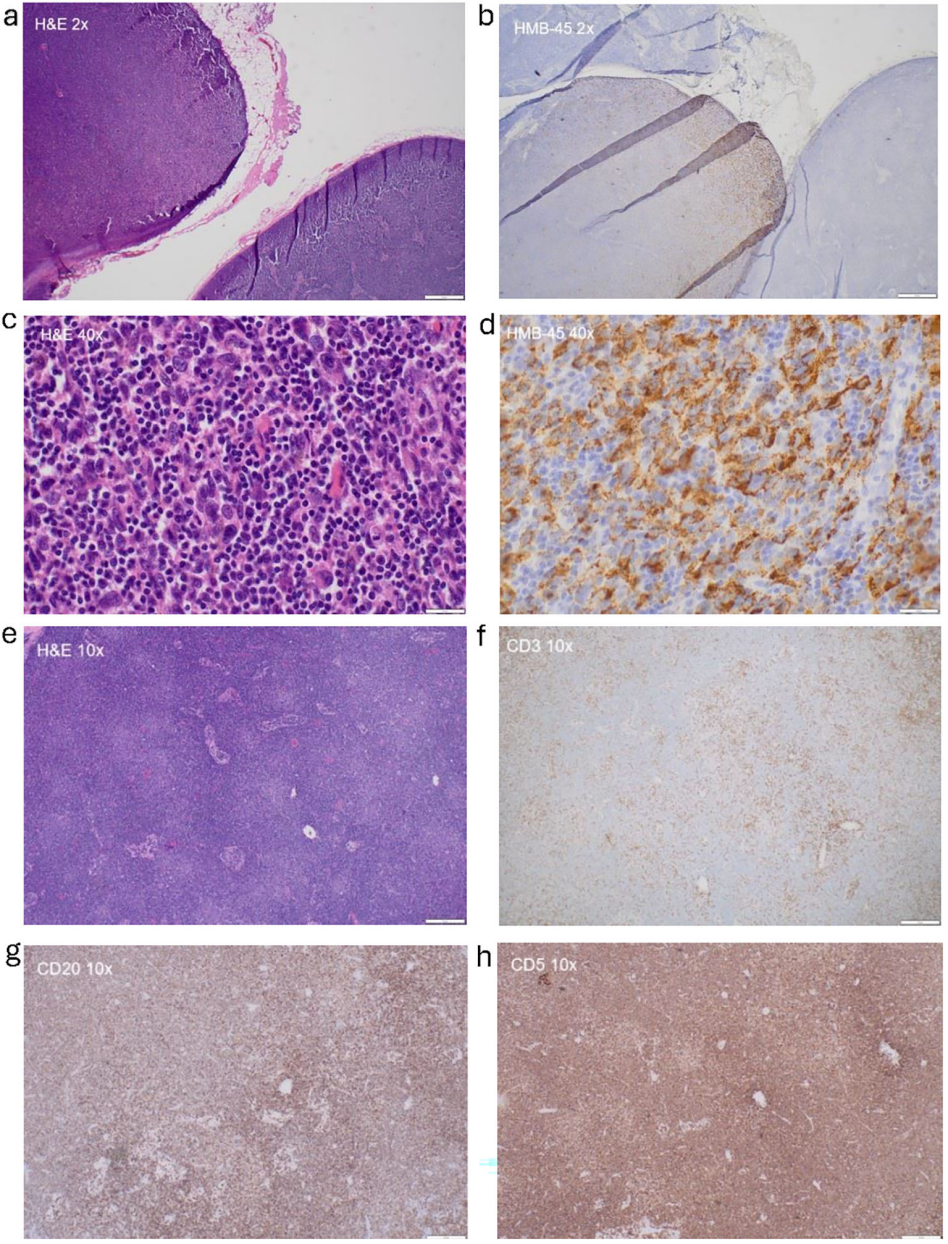
Pathological evaluation of lymph node sample. **(a)** Hematoxylin and eosin (H&E) staining of lymph node shows presence of melanoma and small lymphocytic lymphoma (SLL) (×2). **(b)** HMB-45 staining highlights presence of melanoma in lymph node (×2). **(c)** H&E displays malignant melanoma cells (×40). **(d)** HMB-45 staining shows presence of melanoma in lymph node (×40). **(e)** H&E staining shows presence of SLL (×10). **(f)** CD3 staining highlights background T cells (×10). **(g)** CD20 and **(h)** CD5 staining highlights B cells (×10).

**Figure 2:**
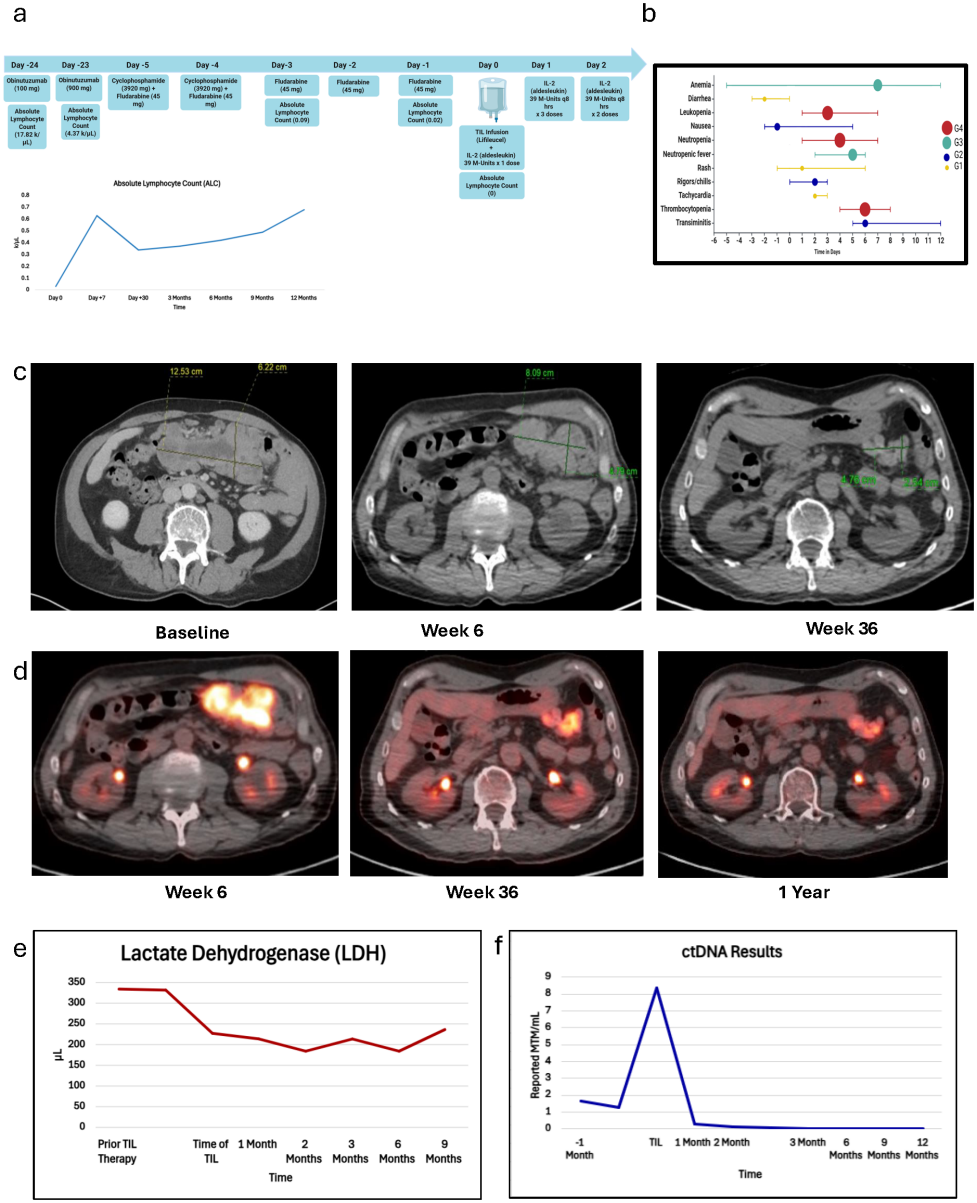
Patient clinical course during tumor-infiltrating lymphocyte (TIL) therapy, evaluating treatment received, adverse events, and response to therapy. **(a)** The administration of drugs, doses, and dates are depicted on the figure. Patient received two doses of obinutuzumab on day −24 and day −23 and thereafter received chemotherapy followed by lifileucel on day 0 and six doses of IL-2 (aldesleukin). **(b)** Treatment-related adverse events. Dots indicate the day of highest toxicity for the adverse event. G1, grade 1; G2, grade 2; G3, grade 3; G4, grade 4. **(c)** CT assessment of target lesion demonstrates decrease in size of mesenteric mass at week 6 and week 36 evaluation timelines. **(d)** PET/CT illustrates metabolic response with decrease in Standardized Uptake Value (SUV) activity of mesenteric mass from SUV max 15.4 at week 6 to SUV max 9.7 at 1 year. Baseline PET/CT was not available for comparison. **(e)** Lactate dehydrogenase (LDH) trend during treatment and follow-up visits. **(f)** Circulating tumor DNA trend during treatment and follow-up visits.

A patient in their 60s was initially diagnosed with pT1a cutaneous melanoma of the chin in December 2015. The patient underwent a wide excision and was on surveillance until February 2021, when they noticed a cervical node enlargement. Fine-needle aspiration (FNA) of the cervical lymph node confirmed the diagnosis of melanoma. The patient underwent left cervical lymph node dissection, which revealed metastatic melanoma (**Figures 1a–d**) in one out of one level 1A lymph node with the largest deposit size of 10 mm and suspicious for extranodal extension, two out of three level 1B lymph nodes being positive for metastatic melanoma with the largest deposit size being 11 mm and the presence of extranodal extension, and two out of twelve level 2 and 3 lymph nodes being positive for metastatic melanoma with the largest tumor deposit size of 1.4 mm and the absence of extranodal extension. An incidental diagnosis was identified when the sections of the lymph nodes (level 1A, level 1B, level 2, and level 3) were further evaluated due to the demonstration of diffuse infiltrate of small mature atypical lymphocytes. Immunohistochemical stains with appropriate controls were performed, showing atypical lymphoid cells showing aberrant positivity for CD20 and CD5, with CD3 highlighting background T cells (**Figures 1e–h**). The findings were consistent with CLL/SLL. Peripheral blood studies demonstrated lymphocytic leukocytosis, and flow cytometry confirmed the diagnosis of CLL in March 2021. The patient received no active treatment for CLL/SLL, as they were asymptomatic and continued with surveillance visits. This was best characterized as Rai stage 0 CLL/SLL, as lymphadenopathy detected on radiological evaluation was deemed mainly due to melanoma as opposed to SLL. For the management of melanoma, the patient continued with 1 year of adjuvant anti-PD-1 therapy with pembrolizumab until January 2022. The patient tolerated treatment well, with the only noticeable side effect being fatigue. Following the completion of treatment, the patient did not have any recurrent melanoma until October 2023, when they developed a peri-gastric mass that was biopsied and confirmed the diagnosis of *BRAF D594N* mutant melanoma. The patient then received two cycles of ipilimumab (3 mg/kg) and nivolumab (1 mg/kg) with restaging scans in December 2023 showing progression of disease with an increase in the size and number of peritoneal lesions along the greater curvature of the stomach, omental disease, and an increase in hilar, axillary, and abdominal nodes. The patient was then switched to nivolumab plus temozolomide in January 2024 as bridging therapy and was referred to Moffitt Cancer Center for evaluation of TIL therapy. Upon referral, the patient underwent vital organ testing, which revealed no abnormal findings, with the only notable finding being lymphocytic leukocytosis (28.8 k/μL) consistent with the known diagnosis of Fluorescence In Situ Hybridization (FISH) normal, TP53 non-mutated, Immunoglobulin Heavy Chain Variable (IGHV) non-mutated, and lymphocyte doubling time >1-year CLL, for which they remained on observation since 2021. Due to this finding, the patient was not eligible for a TIL-based clinical trial. High-resolution HLA typing revealed that the patient was HLA-A*02:01-negative. Considering that the patient was completely asymptomatic and had no immune or infectious complications of CLL along with excellent overall survival outcomes of CLL with a 5-year OS rate of >92% (5), the decision was made to proceed with standard of care lifileucel following FDA approval for the immune checkpoint-resistant metastatic melanoma, which is historically known to be associated with poor overall survival. Further administration of nivolumab and temozolomide was discontinued due to a stable clinical condition, with the overall best response to nivolumab and temozolomide being stable disease. The patient underwent laparoscopic biopsy of the nodular metastatic tissue in the lesser sac, with a tumor implant on the greater omentum chosen as the site for tumor tissue procurement. Given the elevated lymphocyte count and concern for inadequate lymphodepletion after planned chemotherapy before TIL infusion, the decision was made to proceed with CD20-directed cytolytic antibody during the manufacturing period before lymphodepletion (6). The patient received one cycle of obinutuzumab (GAZYVA^®^, Genentech, Inc., South San Francisco, CA, USA), which resulted in the normalization of total leukocyte count to 7.11 k/μL pre-lymphodepletion on day −6. They then received a standard non-myeloablative lymphodepletion chemotherapy regimen with cyclophosphamide (60 mg/kg on days −5 and −4) and fludarabine (25 mg/m^2^ on days −5, −4, −3, −2, and −1) followed by lifileucel (on day 0) and six doses of IL-2 at a dose of 600,000 IU/kg every 8 hours (**Figure 2a**). Due to the risk of infections associated with B-cell depletion, the diagnosis of CLL, and lymphodepletion, immunoglobulin (Ig) G levels were checked both at baseline before proceeding with TIL infusion and longitudinally after TIL infusion, demonstrating normal IgG levels (constantly above 800 mg/dL); therefore, no prophylactic Intravenous Immunoglobulin (IVIG) was administered. Per institutional guidelines, the patient was started on antiviral prophylaxis with acyclovir on day −5 and discontinued at the 1-year landmark after completing full zoster vaccination. Fluconazole for antifungal prophylaxis and levofloxacin for antibacterial prophylaxis were initiated during the neutropenia period and continued until Absolute Neutrophil Count (ANC) > 500 cells/mm^3^. Due to the patient’s previous treatment of temozolomide, the patient received a single pentamidine infusion before the initiation of lymphodepletion, and sulfamethoxazole‐trimethoprim was started on day +30 after TIL infusion for *Pneumocystis jirovecii* pneumonia (PJP) prophylaxis. The patient developed neutropenia while being on sulfamethoxazole‐trimethoprim; therefore, PJP prophylaxis was switched to pentamidine and was continued till CD4 > 200 cells/mm^3^ for a total of 8 months.

Treatment-related adverse events included grade 2 chills/rigors responding to hydromorphone, grade 1 rash, grade 1 sinus tachycardia, grade 1 diarrhea, grade 2 nausea, grade 2 non-neutropenic fever, and grade 3 febrile neutropenia. Infectious workup was conducted for non-neutropenic and neutropenic fevers, and all results remained negative for any findings. The patient was empirically treated with piperacillin and tazobactam from day +2 to day +6 and then de-escalated to levofloxacin along with daily granulocyte colony-stimulating factor administration until absolute neutrophil count recovery. Tachycardia and gastrointestinal toxicities were managed by supportive care, including intravenous fluid hydration, antiemetic therapy, and loperamide. Laboratory abnormalities included grade 2 transaminitis, grade 4 leukopenia, grade 4 neutropenia, grade 3 anemia, and grade 4 thrombocytopenia, all recovered without the need for blood product transfusions (**Figure 2b**). At the 6-week response evaluation, the patient demonstrated a radiological partial response by Response Evaluation Criteria In Solid Tumors (RECIST) v1.1 criteria. Follow-up to date at the 12-month assessment showed a continuous radiological response with a 65% decrease of target lesions. A decrease in metabolic activity was observed on a PET scan from 6 weeks and up to 1 year after treatment (**Figures 2c, d**). Serum lactate dehydrogenase (LDH) peaked at 842 U/L on day +7 from a baseline of 290 U/L, then returned to normal (169 U/L) within 1 month of TIL infusion, and continued to be within normal limits at the 9-month follow-up visit (**Figure 2e**). Melanoma circulating tumor DNA (ctDNA) was also collected longitudinally using Natera’s Signatera assay and revealed a value of 1.28 mean tumor molecules (MTM)/mL. Two weeks after TIL infusion, it peaked at 8.38 MTM/mL on day +15, and ctDNA was undetectable 4 months after TIL infusion. The patient remained with a complete molecular response at the twelve-month follow-up visit with complete clearance of ctDNA (0 MTM/mL) (**Figure 2f**). Regarding CLL/SLL status, the patient continued with the follow-up sessions with regular flow cytometry every 3 months and demonstrated no clinical, laboratory, or radiological evidence of recurrent CLL/SLL up to their last 12-month follow-up visit.

### Harvested tumor microenvironment was enriched by CD8^+^ T cells and T-myeloid cell network and contained SLL deposits

**Figure 3:**
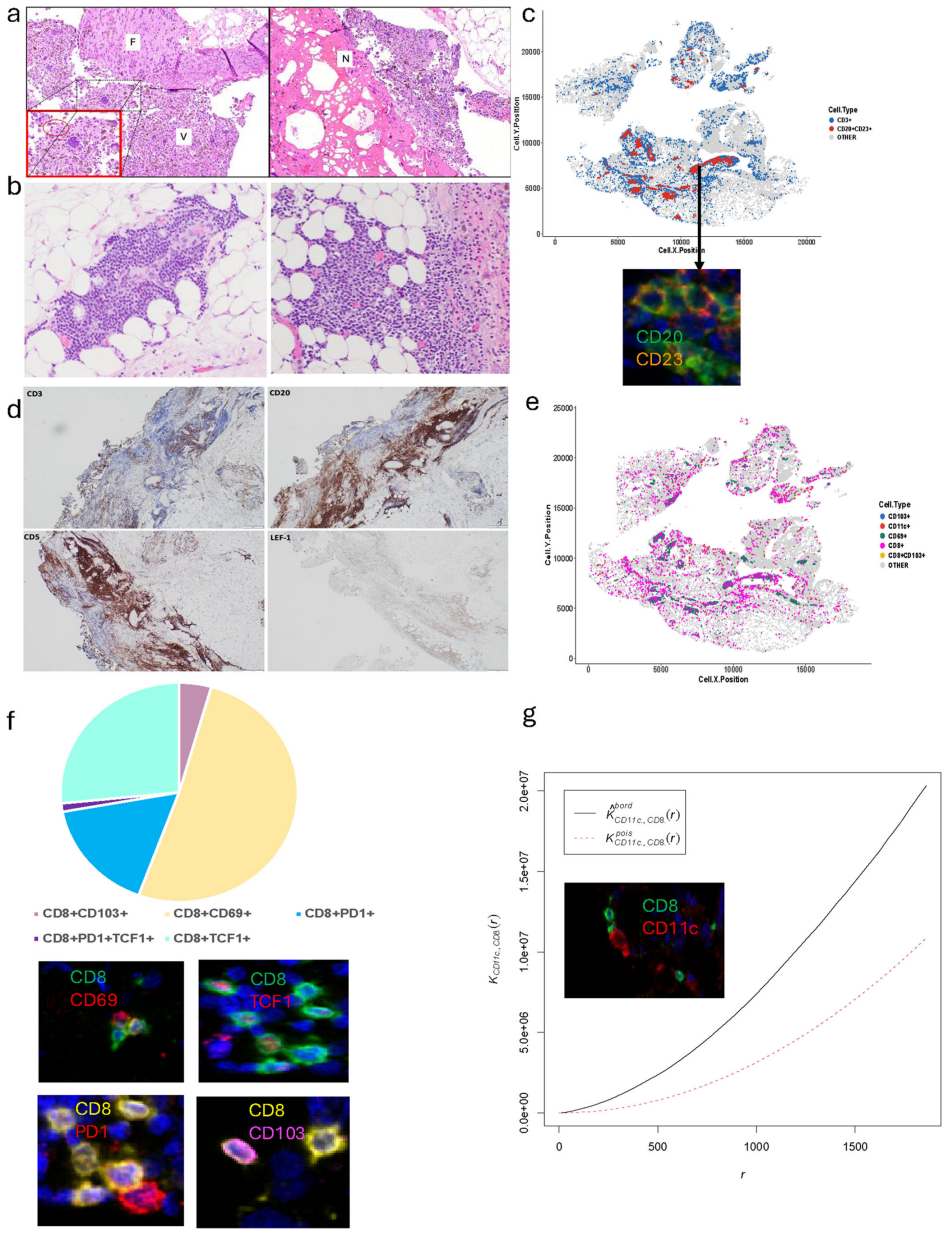
Pathological and immunological assessment of harvested tumor sample. **(a)** Tumor bed assessment using hematoxylin and eosin (H&E) staining reveals presence of viable tumor (V), necrosis (N), proliferative fibrosis (F), and tumor-infiltrating lymphocytes (surrounding viable tumor in inset, magnifying the area highlighted in square and plasma cells in red oval). **(b)** H&E assessment of lymphoid aggregates. **(c)** The cellular composition assessment of lymphoid aggregates using multiplex immunofluorescence (mIF) analysis of cell types such as CD3^+^, CD20^+^, and CD23^+^. **(d)** Single-stain immunohistochemistry assessment of lymphoid aggregates using CD3, CD20, CD5, and LEF-1. **(e)** mIF analysis of cell types such as CD103^+^, CD11c^+^, CD69^+^, CD8^+^, and CD8^+^CD103^+^. **(f)** mIF analysis of cell types such as CD11c^+^CD69^+^, CD8^+^CD103^+^, CD8^+^CD69^+^, CD8^+^PD1^+^, and CD8^+^TCF1^+^. Pie chart represents proportion of CD8^+^CD103^+^, CD8^+^CD69^+^, CD8^+^PD1^+^, and CD8^+^TCF1^+^. **(g)** Quantifying cell colocalization using Cross K function method for an individual case with CD11c^+^ as reference. The black line represents the input image; the red line represents a randomly distributed point pattern. With the increase of radius (X axis), the black line diverges further from the red line, meaning that there is at least one mixed cluster of two types of points, which is also suggested by the positive value of area under the curve (AUC) score for CD11c^+^*vs*. CD8^+^ T cells.

In order to potentially link clinical response to TIL therapy with the pathological and immunological characteristics of the tumor harvested for TIL expansion, the portion of the tumor that was submitted to anatomic pathology was further evaluated. Molecular testing (Moffitt STAR™) showed mutations in *BRAF D594N*, *RAC1*, *TP53*, *LRP1B*, *ATRX*, *CHEK2*, and *NF1*, microsatellite stability, and a high tumor mutation burden of 36.9 mutations/Mb. The histological evaluation of the tumor microenvironment (TME) on H&E revealed 60% viable tumor with 20% necrosis and 20% proliferative fibrosis, along with an abundance of intratumoral tumor-infiltrating lymphocytes and plasma cells (**Figure 3a**). Further assessment of the TME revealed lymphoid aggregates potentially resembling tertiary lymphoid structures (TLSs) (**Figure 3b**), and further phenotyping of those immune structures using our custom-made multiplex immunofluorescence panel revealed CD20^+^CD23^+^ B-cell aggregates (**Figure 3c**). Given the patient’s history of CLL, those B-cell aggregates were further evaluated for potential SLL deposits and demonstrated positivity for CD20, CD5, and LEF-1 (weak positivity), being most consistent with CLL/SLL as opposed to tumor-associated TLS (**Figure 3d**). Those SLL deposits were separated from viable melanoma tissue and mainly located within the surrounding adipose tissue. Next, the TME was assessed using a multiplex immunofluorescence panel for phenotyping of CD8^+^ T cells, which showed an abundance of CD8^+^ T cells along with CD11c myeloid cells (**Figure 3e**). Further phenotyping of CD8^+^ T cells revealed a significant proportion of CD8^+^CD69^+^ tumor-reactive T cells and CD8^+^TCF1^+^ stem-like T cells, followed by CD8^+^PD-1^+^ cytotoxic T cells and CD8^+^CD103^+^ tissue-resident memory T cells (**Figure 3f**). **Supplementary File 1** contains detailed methods of all analyses. Spatial interaction analysis revealed a co-clustering pattern of CD11c myeloid cells with CD8^+^ T cells (**Figure 3g**). In summary, harvested tissue contained a viable melanoma with surrounding pathological findings suggestive of ongoing immune activation, along with CLL/SLL deposits, and was highly enriched by T-myeloid cell network along with CD8^+^CD69^+^ and stem-like T cells.

### TIL product was characterized by CD8^+^ predominant autologous tumor-reactive T cells containing CD39^neg^CD69^neg^ cells and long-term persistence in periphery

**Figure 4:**
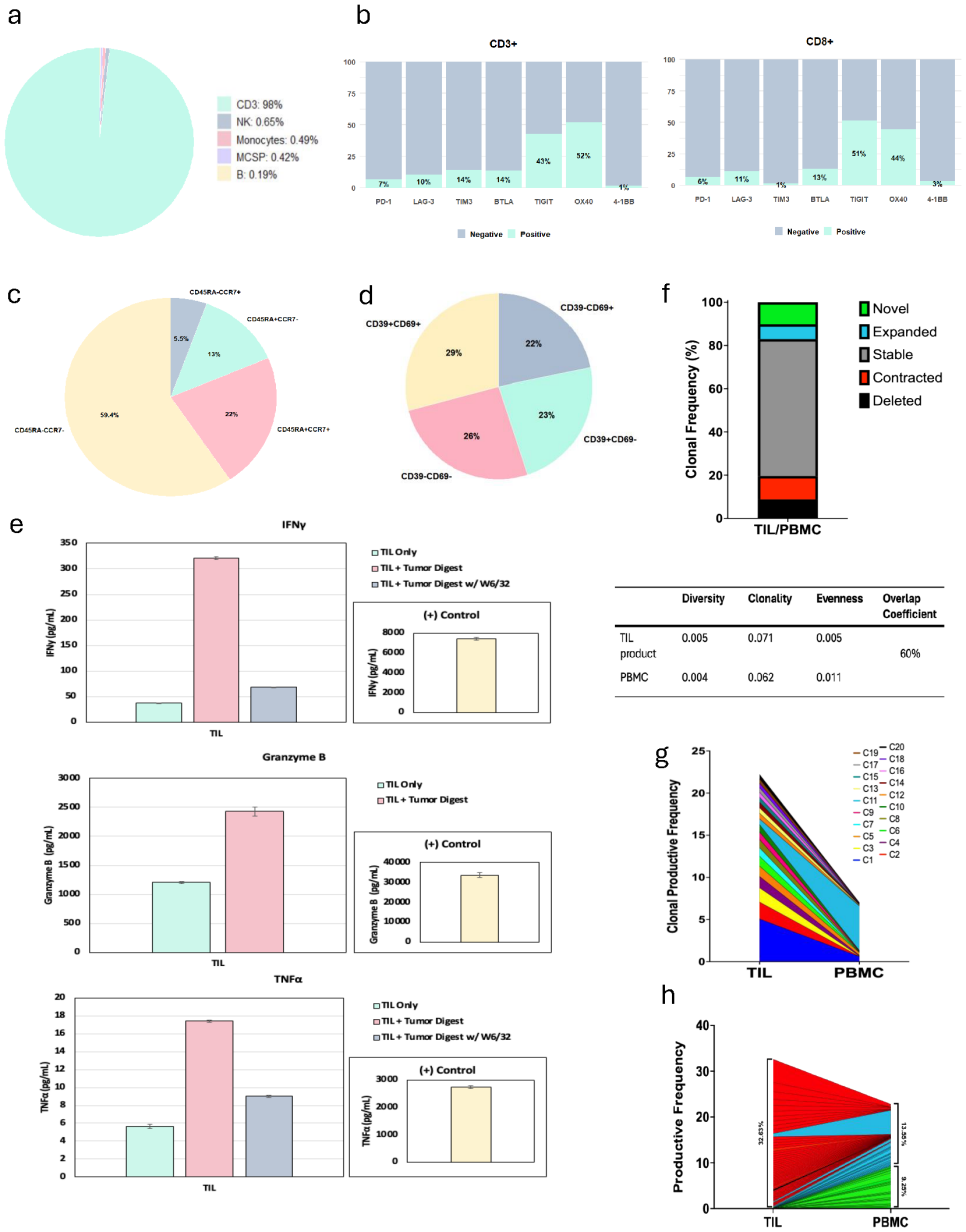
Adoptive cell therapy product phenotypic characteristics, tumor reactivity, and trajectory of T cell clones. **(a)** Pie chart shows immune composition of infusion product; MCSP, melanoma chondroitin sulfate proteoglycan. **(b)** Bar plot shows expression of co-inhibitory and co-stimulatory receptors on total CD3^+^ T cells and CD8^+^ subsets. **(c)** Pie chart shows proportion of effector memory T cells in infused product. **(d)** Pie chart shows proportion of CD39^−^CD69^−^ cells in infused product. **(e)** Co-culture of tumor-infiltrating lymphocyte (TIL) with autologous tumor digest, which demonstrated >60% blocking activity and increase in IFNγ, TNFα, and Granzyme B, confirming TIL reactivity. **(f)** Bar plot shows proportion of novel, expanded, stable, contracted, and deleted clones. Table shows TCRβ diversity, evenness, clonality, and overlap coefficient between TIL product and peripheral blood mononuclear cells (PBMCs). **(g)** Clonal tracking by TCRβ sequences of the top 20 infused TIL clones. **(h)** Productive frequency of top 50 clones in TIL product (32.63%), shared clones between TIL and PBMCs (13.55%), and new clones in PBMCs (9.25%).

To evaluate whether the TIL product contained any significant amount of CLL cells, we requested manufacturing information from Iovance Biotherapeutics, which revealed a total viable cell count of 15 × 10^9^, a viability of 89% with 98% of CD3^+^ T cells, and only a negligible amount of B cells (**Figure 4a**). To further determine the characteristics of the TIL product potentially contributing to the clinical response in our case, we performed flow cytometry on TIL recovered from the product infusion bag after the completion of infusion. The TIL product was mainly composed of CD8^+^ T cells, accounting for 69.5% of total CD3^+^ T cells. We expanded the phenotypic evaluation of total CD3^+^ T cells and CD8^+^ subsets by interrogation of the surface expression of co-stimulatory and co-inhibitory receptors (**Figure 4b**). The results showed OX40 and TIGIT being the highest expressed on CD8^+^ T cells, followed by BTLA and LAG3, with only 6.5% being CD8^+^PD-1^+^ cells. As anticipated, infused TIL had mainly CD45RA^neg^CCR7^neg^ effector memory phenotype while notably containing 22% of CD45RA^+^CCR7^+^ naïve T cells within CD8^+^ T cells (**Figure 4c**). Importantly, 25.9% of total CD8^+^ T cells were CD39^neg^CD69^neg^, a stem-like population of CD8^+^ T cells that has been previously identified as being associated with response to TIL therapy (**Figure 4d**). Next, to evaluate TIL activity, we co-cultured TIL alone, with autologous tumor digest, or with autologous tumor digest treated with W6/32 antibody to block MHC class I. Increased IFNγ, TNFα, and Granzyme B were measured after a 24-hour co-culture, which was decreased with an MHC class I blocking antibody, confirming MHC class I-directed TIL reactivity against autologous tumor (**Figure 4e**). To evaluate whether TIL present in the infusion product were also reactive toward CLL antigens, we first isolated CD5^+^CD19^+^ B cells from peripheral blood mononuclear cells (PBMCs) collected at the time of tumor harvest and before obinutuzumab administration. Notably, CD19^+^ cells accounted for 83.6% of live cells, and 99.8% of those were also CD5^+^, representing CLL (**Supplementary Figure 1**). Next, we co-cultured TIL alone, with CD5^+^CD19^+^ B cells, or with CD5^+^CD19^+^ B cells treated with W6/32 antibody to block MHC class I, and the results revealed no TIL reactivity to CLL (**Supplementary Figure 2**).

Finally, we interrogated TIL clonotype and persistence through the performance of TCRβ complementarity-determining region (CDR) sequencing of infusion product and PBMCs collected at 9-month follow-up visit. There was a 60% overlap of TCRβ CDR sequences between the infusion product and PBMCs, suggestive of strong persistence of the infusion product in the periphery (**Figure 4f**). While most individual clones from the TIL product remained stable in the periphery, the PBMCs at 9 months also demonstrated the expansion of unique clones as well as the contraction of other individual clones (**Figure 4g**). When further examining clonal repertoire, the top 50 clones accounted for ~1/3 of total clones within the infusion product, demonstrating the polyclonal nature of the product (**Figure 4h**). Altogether, the infusion product for this patient demonstrated superior quality due to factors such as CD8^+^ TIL predominance, tumor-specific reactivity, the presence of stem-like CD39^neg^CD69^neg^CD8^+^ T cells, and a strong overlap of TCRβ sequences between infusion product and periphery, likely contributing to durable TIL response.

## Discussion

Patients with CLL are at an increased risk of developing both melanoma and non-melanoma skin cancers (7). Notably, CLL is associated with worse melanoma-specific survival (8) and leads to immune dysfunction in both systemic circulation and the local TME, targeting both innate and adaptive immune cell subsets (9). T cells from CLL patients are characterized by an increased expression of PD-L1, cytotoxic T-lymphocyte-associated protein-4 (CTLA-4), and other co-inhibitory receptors, exhibiting an exhausted phenotype (10, 11). While large studies are lacking to formally evaluate immune checkpoint blockade (ICB) response rate among patients with concurrent CLL and melanoma, published reports have shown some durable immunotherapy responses in those patients with acceptable safety profiles (12, 13). Successful performance of TIL therapy in patients with advanced melanoma and concomitant CLL/SLL may be challenging due to the potential contamination of the harvested tumor sample with SLL and IL-2-induced *ex vivo* modulation of atypical B lymphocytes (14). Early experimental studies have revealed that IL-2 may serve as a survival factor for B-cell CLL (B-CLL) through the enhancement of antiapoptotic proteins (15), although high-dose IL-2 has been investigated as a therapeutic strategy for patients with CLL (16). Furthermore, patients with CLL have hypogammaglobulinemia along with the risk of infections and autoimmune adverse events, which should be taken into account when following up patients after lymphodepleting chemotherapy. When assessing patients with CLL and melanoma, it is important to appreciate that ICB-refractory melanoma has limited therapeutic options and poor survival outcomes, while stage 0 CLL is an indolent disease with excellent overall survival, often not requiring immediate treatment. Therefore, in our case, the decision was made to proceed with TIL therapy despite potential challenges. This case is the first to report the feasibility and efficacy of TIL therapy in a patient with advanced melanoma and concurrent CLL/SLL. The treatment was well tolerated and resulted in no new adverse events other than those anticipated with chemotherapy, TIL, and IL-2. While the patient received no previous treatment for CLL, we opted to treat with an anti-CD20-directed antibody to achieve optimal lymphodepletion along with the administration of non-myeloablative chemotherapy, which resulted in absolute lymphocyte count (ALC) of 0. In this case study, the patient demonstrated a radiological response to TIL therapy and had a molecular response with complete clearance of ctDNA. Notably, there was an initial transient spike in ctDNA at 2 weeks after treatment, likely reflective of tumor cell death, followed by complete clearance of ctDNA.

Pathological response to immune checkpoint blockade therapy in the neoadjuvant setting is an established way of assessing treatment response. Historically, a major pathological response has been characterized by the absence of viable tumor along with the presence of necrosis and fibrosis and is correlated with improved survival outcomes (17). The pathological response assessment in metastatic melanoma core biopsies has also been evaluated and has been associated with improved overall survival outcomes in patients with major pathological response on biopsy (18). Our pathological assessment of the harvested tumor sample revealed that while the patient had >50% viable tumor present in the sample, necrosis, proliferative fibrosis, TIL, CLL/SLL hubs, and plasma cells were also measured, potentially indicating an ongoing local immune response. The proportion of viable tumor and surrounding fibroinflammatory stroma in the procured tumor tissue may be of importance for successful TIL expansion and requires further studies. The multiplex immunofluorescence (mIF) studies further revealed the presence of stem-like CD8^+^ T cells being a niche for sustaining T cells in the TME, as well as CD8^+^-myeloid co-clustered networks, which have previously been described to be associated with TIL response (19, 20). While the harvested tumor had SLL deposits, it resulted in a CD8^+^ predominant, tumor-reactive TIL product with an increased expression of LAG-3 and TIGIT. Our previous report showed an increased surface expression of LAG-3 and TIGIT on CD8^+^ T cells in TIL responders (21). The infusion product also contained CD39^neg^CD69^neg^ CD8^+^ T cells, likely contributing to long-term TIL persistence and durable anti-tumor efficacy (22). While the majority of clones were stable in the TIL product and PBMCs at the 9-month follow-up visit, the TIL product appeared to be polyclonal, with the top 50 clones accounting for approximately 1/3 of total clones. Notably, preferential expansion was observed in a few clones in PBMCs, and further characterization of those clones is warranted.

In summary, despite the challenges posed by concomitant diagnoses of CLL/SLL and metastatic melanoma in a patient receiving TIL therapy, this case underscores its potential as a treatment option for patients with immune checkpoint-resistant/refractory advanced melanoma and concurrent stage 0 CLL/SLL. The management of these patients should be considered on a case-by-case basis with multidisciplinary discussion at the tumor board. Importantly, TIL therapy may introduce more risk for higher-stage CLL patients, particularly for those with CLL-associated hematologic complications such as autoimmune hemolytic anemia or thrombocytopenia; therefore, at this time, our findings should not be generalized to patients with other than stage 0 CLL/SLL. As more patients are treated, further studies will report the safety and efficacy for larger patient cohorts as well as immunological correlates of response.

## Data Availability

The original contributions presented in the study are included in the article/**Supplementary Material**. Further inquiries can be directed to the corresponding authors.
