# Supplementary material for: Case Report: Durable response to tumor-infiltrating lymphocyte therapy in a patient with metastatic melanoma and chronic lymphocytic leukemia/small lymphocytic lymphoma
Source: Front Immunol. 2025 Nov 17;16:1718443. doi: 10.3389/fimmu.2025.1718443 (PMC12665918; doi:10.3389/fimmu.2025.1718443)
Supplement: Supplementary file 1 [file DataSheet1.pdf]

Supplementary figure 1: Gating strategy for chronic lymphocytic leukemia cells. Representative flow plot for CD19 and CD5 gating strategy.

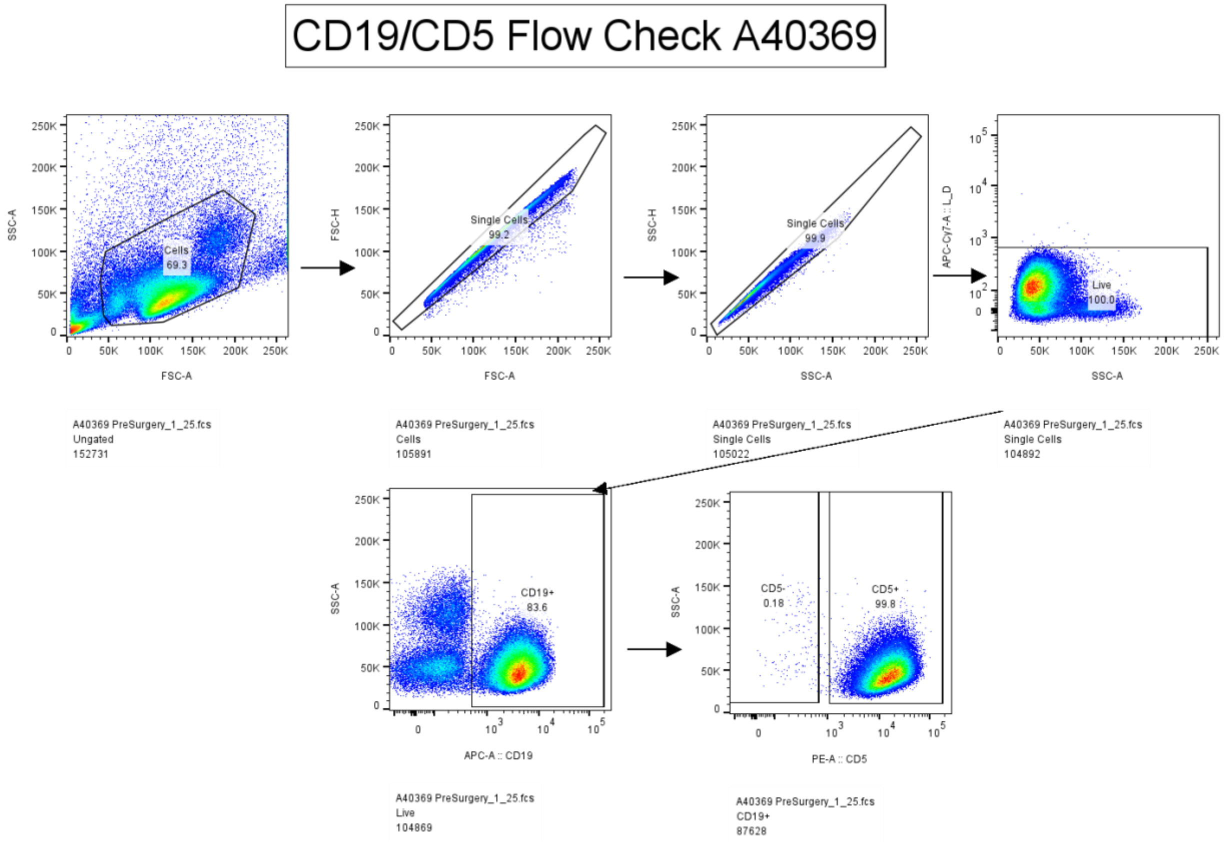

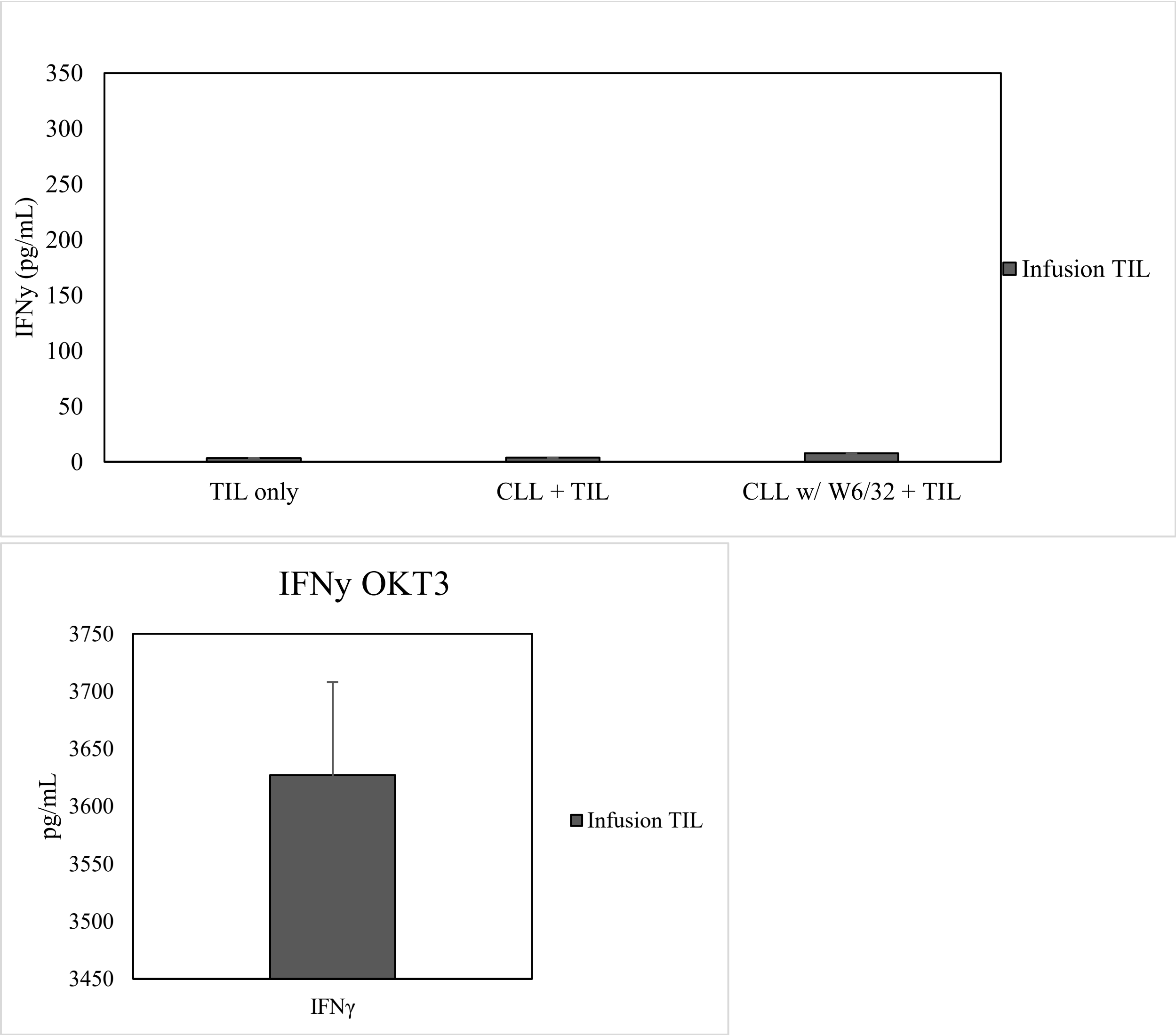

Supplementary figure 2: TIL co-culture with CLL showing no reactivity of TIL to CLL.
