# Supplementary material for: Case Report: Durable response to tumor-infiltrating lymphocyte therapy in a patient with metastatic melanoma and chronic lymphocytic leukemia/small lymphocytic lymphoma
Source: Front Immunol. 2025 Nov 17;16:1718443. doi: 10.3389/fimmu.2025.1718443 (PMC12665918; doi:10.3389/fimmu.2025.1718443)
Supplement: Supplementary file 2 [file DataSheet2.pdf]

Multiplex Immunofluorescence Analysis

Formalin-fixed, paraffin-embedded (FFPE) tissue sections were stained using the Opal™ 7-Color Automation IHC Kit (Akoya Biosciences, Waltham, MA) on the BOND RX autostainer (Leica Biosystems, Vista, CA). The Opal system employs tyramide signal amplification (TSA)-conjugated fluorophores to detect multiple targets within a single assay. Sections were baked at 65 °C for 2 hours and subsequently processed on the BOND RX. All steps, including deparaffinization and antigen retrieval, followed the automated Opal IHC protocol (Akoya). Heat-induced epitope retrieval (HIER) was performed using EDTA buffer (pH 9.0) at 95 °C for 20 minutes, followed by blocking with Akoya blocking buffer for 10 minutes.

Multi-layer TIFF images were exported from inForm (Akoya) and analyzed using HALO software (Indica Labs, Corrales, NM). A machine-learning classifier was trained to distinguish tumor, stromal, and non-tissue regions, with pan-cytokeratin serving as the tumor mask. Cell segmentation was performed using DAPI nuclear staining. Positivity thresholds for each marker were determined based on published staining patterns and fluorescence intensity. Quantitative outputs included positive cell counts and percent positivity per marker, with optional per-cell data export containing classification and fluorescence intensity metrics.

Reagent	Lot#	Product#	Opal	Clone	Dilution	Manufacturer	AR
Target Antibody 1 ***CD103***	1031913-1	ab227697	OPAL-520		**1:150**	Abcam	**ER2**
Target Antibody 2 ***TCF1/7***	11	2203s	OPAL-620		**1:75**	CST	**ER2**
Target Antibody 3 ***CD8***	41627944	M7103	OPAL-690		**1:50**	Dako	**ER2**
Target Antibody 4 ***PD1***	5	86163s	OPAL-570		**1:100**	CST	**ER2**
Target Antibody 5 ***CD69***	GR3280305-15	ab233396	OPAL-480		**1:100**	Abcam	**ER2**
Target Antibody 6 ***CD11c***	GR3450269-6	ab52632	OPAL-780		**1:75**	Abcam	**ER2**

Reagent	Lot#	Product#	Expiration Date	Clone	Dilution	Manufacturer	AR
Target Antibody 1 ***CD23***	WF3304963	MA5-14572	OPAL-620	SP23	**1:100**	Invitro	**ER2**
Target Antibody 2 ***Ki67***	1015296-27	ab16667	OPAL-480	[SP6]	**1:75**	Abcam	**ER2**
Target Antibody 3 ***CD20***	41597660	M0755	OPAL-690	L26	**1:200**	Dako	**ER2**
Target Antibody 4 ***pNAD***	4023532	MABF2050	OPAL-570	MECA-79	**1:200**	Sigma	**ER2**
Target Antibody 5 ***CD3***	41654191	A0452	OPAL-520	Rb poly	**1:200**	Dako	**ER2**
Target Antibody 6 ***CD21***	1006248-4	ab75985	OPAL-780	[EP3093]	**1:300**	Abcam	**ER2**

Flow Cytometry

Tumor-infiltrating lymphocytes (TILs) were washed with PBS and stained with Live/Dead Fixable Near-IR dye (BioLegend) according to the manufacturer's protocol. Peripheral blood mononuclear cells (PBMCs) were washed with sterile Flow Buffer (FB; 5% FBS, 1 mM EDTA, 0.1% sodium azide in PBS) and blocked with FcR Blocking Reagent (Miltenyi Biotec) at 4 °C. TILs were stained with HLA-A*03:01 gp100 APC tetramer (MBL Life Science) on ice for 60 minutes, protected from

light. Flow acquisition was performed using a BD FACS Celesta (BD Biosciences), and data were analyzed with FlowJo software.

We included combinations of markers (CD3, CD4, CD8, PD-1, BTLA, 4-1BB, OX40, TIGIT, TIM3, CD39, CD69, Ki67, CCR7, CD45RA, and CD103) labeled with fluorophores from BioLegend and BD Biosciences, as detailed in below tables.

Marker	Fluorophore	Catalog	Vendor
L/D	Zombie NIR		
CD3	PerCpCy5.5	300328	BioLegend
CD8	FITC	300906	BioLegend
CD69	BV510	310936	
CD39	BV605	328236	BioLegend
Ki67	APC	350514	BioLegend
CCR7	BV786	566758	BD Biosciences
CD45RA	Pacific Blue	304123	BioLegend
CD103	PE	260330	BioLegend

Marker	Fluorophore	Catalog	Vendor
L/D	Zombie NIR	423106	BioLegend
CD3	PerCpCy5.5	300328	BioLegend
CD4	FITC	300906	BioLegend
CD8	BV510	300934	BioLegend
PD-1	BV421	329920	BioLegend
BTLA	BV650	369316	BioLegend
4-1BB	APC	309804	BioLegend
OX40	PECy7	350012	BioLegend
TIGIT	PE	372704	BioLegend
TIM3	BV605	119721	BioLegend
Tetramer Staining	Flurophore	Catalog	Vendor

HLA-A*03:01	APC	TC-0137-2	MBL Bio
Marker	Fluorophore	Catalog	Vendor
L/D	Zombie NIR	423106	BioLegend
CD5	PE	300608	BioLegend
CD19	APC	302212	BioLegend

Reactivity Assay

Samples expressing an HLA-A2 phenotype were tested for tumor reactivity using HLA-matched melanoma cell lines (WM35, 526, SBLC2, 266-4, and 239A). Co-cultures were prepared in 96-well plates (1 × 10^5 cells/well for both TILs and tumor cells) under four conditions: (1) OKT3 (0.5 μg/mL), (2) TIL only, (3) TIL + HLA-matched cell line, and (4) TIL + HLA-matched cell line + HLA-A,B,C blocking antibody (W6/32; 10 μg/mL). After overnight incubation, supernatants were collected and analyzed for IFN-γ using the ELLA platform (Bio-Techne, catalog #SPCKB-PS-002574). TILs producing >100 pg/mL IFN-γ with ≥70% blocking were classified as tumor-reactive.

CLL / T-cell Co-culture Assay

PBMCs were stained with Live/Dead Fixable Near-IR dye (BioLegend) and blocked with FcR Blocking Reagent (Miltenyi Biotec). Cells were surface-stained with CD19 APC (BioLegend, 302212) and CD5 PE (BioLegend, 300608) for 20 minutes at 4 °C, protected from light. After washing, cells were resuspended in PBS containing 5% human serum (HS) and sorted using a Cytek Aurora CS Cell Sorter (Cytek Biosciences) into $CD19^+CD5^+$ (CLL B cells) and $CD19^-CD5^-$ (non-B cells) populations.

Sorted PBMCs and TILs were co-cultured at a 1:1 ratio (1 × 10^5 cells each per well). Positive controls were established by pre-coating wells with OKT3 (30 ng/mL, 1 hour). Blocking antibody HLA-A,B,C W6/32 (BioLegend, 311402) was added 30 minutes before co-culture at a final concentration of 10 μg/mL. Cultures were incubated for 24 hours at 37 °C, 5% CO_2, and supernatants analyzed for IFN-γ, TNF-α, Granzyme B, and IL-2 using the Ella immunoassay (Bio-Techne, SPCKC-CS-003222).

TCR Sequencing

DNA was extracted from TIL and PBMC samples using the DNeasy Blood & Tissue Kit or AllPrep DNA/RNA Mini Kit (Qiagen Sciences) and quantified using a NanoDrop spectrophotometer (Thermo Fisher Scientific). T-cell receptor beta (TCRβ) sequencing was performed using the ImmunoSEQ® TCRβ Kit v3 or v4 (Adaptive Biotechnologies, Seattle, WA, USA) at the Adaptive Biotechnologies ImmunoSEQ Laboratory. Data were analyzed using the ImmunoSEQ Analyzer v3.0 to assess shared TCRβ CDR3 amino acid sequences.
